# Science, Technology and Innovation as Social Goods for Development: Rethinking Research Capacity Building from Sen’s Capabilities Approach

**DOI:** 10.1007/s11948-018-0037-1

**Published:** 2018-03-01

**Authors:** Maru Mormina

**Affiliations:** Faculty of Humanities and Social Sciences, University of Winchester, Winchester, UK

**Keywords:** Development, Capacity building, Science technology and innovation, Research systems, North–South partnerships, Capabilities approach, Collective or social capabilities, Low and middle income countries

## Abstract

Science and technology are key to economic and social development, yet the capacity for scientific innovation remains globally unequally distributed. Although a priority for development cooperation, building or developing research capacity is often reduced in practice to promoting knowledge transfers, for example through North–South partnerships. Research capacity building/development tends to focus on developing scientists’ technical competencies through training, without parallel investments to develop and sustain the socioeconomic and political structures that facilitate knowledge creation. This, the paper argues, significantly contributes to the scientific divide between developed and developing countries more than any skills shortage. Using Charles Taylor’s concept of irreducibly social goods, the paper extends Sen’s Capabilities Approach beyond its traditional focus on individual entitlements to present a view of scientific knowledge as a social good and the capability to produce it as a social capability. Expanding this capability requires going beyond current fragmented approaches to research capacity building to holistically strengthen the different social, political and economic structures that make up a nation’s innovation system. This has implications for the interpretation of human rights instruments beyond their current focus on access to knowledge and for focusing science policy and global research partnerships to design approaches to capacity building/development beyond individual training/skills building.

## Introduction

The ability to generate scientific and technological knowledge (S&T) and translate it into new products or processes is a key instrument of economic growth and development. Yet, S&T, and societies’ capacity to produce it, is unequally distributed. In light of this inequality, this article offers a normative analysis on the global distribution of S&T capacity. The purpose is two-fold: first, to outline an ethical framework for evaluating different arrangements for the creation and sharing of scientific knowledge globally; and second, to inform policy and funding strategies for developing scientific capacity in low and middle-income countries (LMIC).^1^

Lofty aspirations to *enhance scientific research and upgrade the technological capabilities* of LMIC (UN 2015) has led to the adoption of research capacity building (RCB)^2^ as a cornerstone of international development assistance (Colglazier 2015). Yet, concrete efforts to empower these countries to develop their capacity to produce rather than consume knowledge have been piecemeal. This is unsurprising, given that the concept of *capacity* remains under-theorised and open to diverse interpretations. Current understandings of capacity are largely Western-centric and rooted on ideas borrowed from disciplines such as performance management, and organisational development (Morgan 2006), but also from left-leaning ideals of empowerment, participation and community development (Eade 1997). Yet, a clear definition of what constitutes capacity is missing, and consequently, also the frameworks that can help with its assessment, management, monitoring and evaluation (Morgan 2006). As a result, practitioners vary considerably in their approaches to capacity building: for some it is as a pure human resources issue, whereas for others is about organisational change and general management. And whilst most international development organisations espouse the basic principle of capacity building as empowerment, in practice it is often operationalised as a means to solve practical problems *(ibid).*

Lack of conceptual clarity is also seen in donors’ approaches to RCB. Research investments in LMIC aim primarily to the production of research outputs (Enoch 2015), often by high income countries (HIC) teams in collaboration with LMIC researchers. RCB is often seen as an ethical requirement to level the playing field between collaborators with unequal capacities and resources for research (Parker and Kingori 2016), and thus the focus is strongly on skills development of local scientists. This approach to RCB is popular because it is easier to implement, measure and evaluate, but gives insufficient attention to the wider and long term social factors that help or hinder local knowledge production. Yet, if science is to be harnessed to promote social and economic progress in LMIC, RCB must be viewed as integral to development strategies and approached more holistically at a macro, systems level, not just at a micro, individual level. However, taking such a systems approach begs some normative questions: What is required to build scientific capacity? And whose capacities need to be built?

Efforts to address these questions have been sparse. Some have attempted to link S&T to human rights instruments, specifically Article 27 of the Universal Declaration of Human Rights (UDHR) and Article 15 of the International Covenant on Economic, Social and Cultural Rights (ICESCR), which promote universal access to scientific research and its benefits (Shaver 2010; Plomer 2013; Chapman and Wyndham 2013). These articles have been used to advance two basic ideas: first, that in order to enhance LMIC’s capacity to use S&T for development, these countries must be able to fairly access and benefit from existing knowledge. This creates entitlements to education, access to scientific publications, the promotion of scientific cooperation and international exchanges (Shaheed 2012), as well as the lifting of intellectual property (IP) restrictions to utilise knowledge (Shaver 2010). Here, the focus is on knowledge transfers from HIC to LMIC with little consideration of the latter’s systemic ability to utilise knowledge, and the relevance of such knowledge to LMIC’s specific needs. Second, that scientific capacity is a good to be distributed to individuals alone. This reduces RCB to strengthening scientists’ technical competencies through education and training without parallel investments to develop and sustain the structures wherein knowledge is created. Thus, whilst the human rights language has the *normative teeth* to impact upon priority setting and resource allocations decisions by the international community, its usefulness to solve the capacity problem in LMIC is less clear.

This article moves away from discourses of access and knowledge transfers by positing RCB as a tool for knowledge creation. It then makes a normative case for a system approach to RCB built upon a notion of scientific knowledge as a *social good*, i.e. a good that can only be possessed by and benefit society as a whole. It considers the distribution of this social good (and the capability to produce it) through the lens of the Capability Approach (CA) (Sen 2001), expanded to include societies and their institutions. This helps focus not only on the interests of scientists and beneficiaries of the scientific enterprise (patients, consumers, etc.) but on the social structures and processes necessary for the creation and utilisation of knowledge, i.e. on systems. Therefore, an entitlement to a social capability to produce scientific knowledge requires expanding the scope of RCB from a mere empowerment of individuals to the strengthening of the social, political and economic structures that constitute the scaffolding of a nation’s research and innovation system.

The first part of this article critiques traditional understandings of scientific knowledge as an *asset,* distributed to and possessed by individuals, which in turn determine normative stances and practical approaches to RCB. The second part moves the focus of analysis from *knowledge assets* to *knowledge capabilities*, using Sen’s Capability Approach (CA) as a framework. The article argues that scientific knowledge cannot be construed as an *individual good* in the traditional Senian view (a feature *of* and valuable *to* individuals) but as a *social good* (a feature *of* and valuable *to* societies as a whole). If scientific knowledge is a social good, the capacity to produce it is a *social capability* that emerges from and depends on the social and institutional structures that constitute the condition for knowledge creation. It concludes by briefly examining the implications of this move, outlining how societies’ claim to a capability for producing scientific knowledge requires a re-conceptualisation of RCB as a multilevel approach to strengthen research systems and the societies in which they are embedded.

## Scientific Knowledge as an *Asset* and Research Capacity as *Individual*-*Centred*

Knowledge (of which scientific knowledge is a particular type) is mostly discussed in the knowledge management literature as the intellectual capital of organisations, with a focus on the processes involved in its production (Rowley 2007). Much of this theoretical work builds upon the Data–Information–Knowledge–Wisdom hierarchy (DIKW), a widely recognised model credited to Russell Ackoff (1989). DIKW describes a tiered albeit fluid relationship between data, information, knowledge and wisdom: data refers to elements of an observation or recorded descriptions that are disorganised and therefore have no meaning; data is used to create information (data interpreted and organised to convey meaning); information is used to create knowledge and knowledge is used to create wisdom (Rowley 2007). Knowledge and wisdom are less well defined, though the former seems to refer to a process of accumulated learning internal to the individual that results from synthesising information from various sources and over time (Keri et al. 2006). Wisdom is hardly discussed in the literature (Rowley 2007) but implies the application of judgement upon knowledge in order to guide action. Despite lack of consensus on the above definitions and the processes that convert one into the other (Frické 2009; Zins 2007), the DIKW model is useful to highlight data, information and knowledge as distinct constructs. This distinction is, as explained below, not only semantic but normative. This article is not concerned with a capability to use, produce or share scientific data or information, as this has been done by others (e.g. Bezuidenhout et al. 2017) but a capability to generate scientific knowledge, here understood as a process of synthesis and accumulated learning based upon open, systematic and objective empirical observations of the world.

Scientific knowledge underpins much of the technological capacities that fuel the knowledge economy: *production and services based on knowledge*-*intensive activities that contribute to an accelerated pace of technological and scientific advance* (Powell and Snellman 2004). Because of this increased reliance on intellectual capabilities for wealth production, scientific knowledge is considered an intangible *asset* necessary for development. Countries must be able to use and exploit knowledge to drive social and economic progress, and this in turn determines approaches to RCB largely aimed at transferring knowledge at the micro level of the individual, a focus the following sections seek to challenge.

### Intangible Assets: Consuming Information or Producing Knowledge?

In orthodox economic theories, scientific knowledge is considered a key intangible asset that drives economic development within a country (Romer 1986) and reduces technological and economic differences between countries (Abramovitz 1986), thus, a private good to be appropriated and commercialised for economic benefit *(ibid).* Yet, it, can also be said to be a public good, whose use is non-rivalrous (all can use it) and non-excludable (use by one actor does not preclude other actors from benefitting) (Stiglitz 1999). This framing has been used to spur action to bridge the knowledge divide between countries by ensuring universal access to essential knowledge and technologies, through initiatives such as IP reforms (Shaver 2010), open science/data sharing (Contreras 2010), or open access publishing (Chan and Costa 2005). A closer scrutiny of these discourses, however, suggests that what is often referred to as *knowledge* aligns more closely with the definitions of *data* and *information* given above. In fact, the knowledge assets contained in patents or scientific publications is a particular type of *existing* knowledge that can be expressed and shared through formal language, i.e. *codified* (Polanyi 1966) which, because it is external to the agent’s cognitive processes of decoding and interpreting, some argue is simply *information* (Johnson et al. 2002). This type of codified knowledge/information is often assumed to be relevant and applicable to the needs of developing countries and directly transferrable to these contexts (Chan and Costa 2005). This is not always the case, as scientific knowledge and derived technologies are purpose-driven and context-dependent (Fu et al. 2011). As the bulk of the world’s scientific output is produced by scientists associated with institutions in HIC (Mazloumian et al. 2013) and in response to the specific needs of those nations, access to knowledge by LMIC is not straightforward. It requires developing absorptive and adaptive capabilities necessary for its acquisition and subsequent translation into technologies adapted to local conditions.

Thus, construing scientific knowledge as an asset can result in a limited focus on simply transferring and consuming information, a process not always costless. Undoubtedly, facilitating access to scientific information is important and necessary to propel technological development, particularly in the case of innovations entailing leapfrogging, but not sufficient. Much of the knowledge that underpins the innovation process is *tacit*, not easily embodied, codifiable or readily transferable (Polanyi 1966). Tacit (or informal) knowledge is unwritten, unspoken, and based on individuals’ experiences, intuitions, observations, internalised information, and above all interactions. It is this kind of knowledge that results in new or improved products, operational processes or approaches to a social service, i.e. innovation (Frascati-Manual 2015). Codification makes *existing knowledge/information* easily transferrable; yet, in the process, the less tangible aspects of knowledge are lost, and for this reason *new tacit knowledge* is less mobile. This explains the concentration of knowledge generation capabilities around innovation *hubs*, and the widening knowledge divide between HIC and LMIC despite globalisation’s erosion of borders and the pervasiveness of information technologies (Gertler 2003).

The importance of innovation rarely comes under the scrutiny of global theories of justice, and when it does, the moral discourse appears more concerned with the distribution of the products of S&T innovation rather than the capacity to innovate (Papaioannou 2011, 2014). This capacity depends not only on the ability to absorb codified information but crucially to produce tacit forms of knowledge. This ability remains largely within the domain of HIC (Mazloumian et al. 2013), particularly with regards to resource intensive knowledge activities (e.g. genomics). This is not a dismissal of the considerable amount of scientific research and innovation that is currently conducted in LMIC; it is the case that a number of them have embraced S&T as a path to development, though many are left behind. If developing societies are not to be excluded from the benefits of S&T, the geographies of knowledge must be rebalanced through approaches that pay enhanced attention to increasing access and exposure to scientific information whilst also fostering homegrown processes and structures that facilitate the production, translation and utilisation of *tacit*, situated forms of knowledge.

### Research Capacity: A Focus on Individuals

A second assumption is that scientific knowledge, whether as a private or public good, must be possessed and exploited by individuals (scientists) and must benefit individuals (patients, consumers, etc.). Justice, situated at the micro level, is achieved when individuals can equitably access and benefit from scientific knowledge, as proponents of the human rights approach discussed earlier would contend. A strong focus on individuals also underpins the position of Timmermann (2014). Grounding his argument in the concept of human capabilities, Timmermann suggests that justice demands not only the distribution of knowledge, possessed and consumed as any other good, but equitable participation by individuals in the co-creation of knowledge. This requires building capacity (of individual scientists) where it is lacking *(ibid).*

Though not all, most approaches to RCB have a strong focus on developing individual skills, perhaps because these are much easier to implement and evaluate (Vallejo and Wehn 2016). RCB is usually delivered through HIC–LMIC partnerships (Velho 2004), which are generally established not for the purpose of developing capacity but of achieving specific scientific goals—Mode 1 model of knowledge production according to the typology of Gibbons et al. (1994). As a consequence, RCB becomes embedded and operationalised within specific scientific projects and often reduced to building the technical competencies of individual scientists, most commonly through education and training. This approach to RCB often assumes a straightforward progression from scientific research (knowledge production) to innovation (its application into new technologies) and to development (economic and/or social): input in the form of trained scientists will result in greater knowledge production, more and better technological innovation and faster development. However, not only is this assumption of linearity unrealistic, it also overlooks the systemic nature of knowledge creation and innovation. The pursuit of scientific knowledge is fundamentally a collective process: individuals use their expertise and collaborate in a highly organised division of labour (within and across research teams) to collect data in meaningful arrangements in order to obtain information and produce the *tacit* forms of knowledge that underpin innovation. This suggests that whilst individuals may *have* scientific knowledge (in the form of accumulated learning), they alone cannot *produce* it (Cheon 2014), but must participate in a complex web of interactions across many different boundaries: disciplinary, geographic, economic. Moreover, downstream translation of scientific knowledge into technological innovation requires further interplays to enable the transfer of knowledge from centres of “knowledge creation” (typically universities) to “centres of knowledge application” (typically industry) where it becomes added value through its embedding into design (of new products, processes or services). It is important to note however, that this is a non-linear relationship with multiple iterations, feedback loops and failures (Lundvall 2007).

Thus, if the process of knowledge creation that underpins innovation rarely takes place outside the specialised formal and informal networks, whether physical or virtual, that constitute a scientific community, then what matters is not so much the strength of individual actors (researchers and research partnerships, universities, firms, markets, governments, etc.) but the connections between them (Velho 2004). From this follows that whilst ensuring equitable access to knowledge and a greater supply of trained scientists are both essential to create a critical mass of expertise in a LMIC, this alone does not generate knowledge. An effective approach to RCB must recognise that the creation of scientific knowledge is bound to the social, economic and political institutions, practices and norms that sustain it, to such extent that the right of individuals to access scientific knowledge and participate in its production (Timmermann 2014) cannot be asserted without also recognising the intrinsic moral importance of the structures where knowledge is created. The production of scientific knowledge is inherently a social phenomenon situated within the complex enmeshing of social, economic and political relations and for this reason cannot be promoted only at the individual level.

In sum, the argument is twofold: first, what matters from the perspective of justice is not only the fair distribution of existing knowledge and technological innovations, but the fair distribution of the capability to produce scientific knowledge and translate it into new products of innovation. Second, this capability is not a capability of individuals but of societies. The first part is, hopefully, uncontentious. After all, this is the conviction underlying the many RCB initiatives which ultimately aim to grow local scientific capacity. However, they do so instrumentally and mostly focusing on individual capabilities without addressing the social context and institutions that condition individuals’ actions and interactions. The contention here is that only by recognising the inextricable connection between science and the social structures from which it emerges can we develop ways to enhance society’s capacity for creating scientific knowledge. And when we do that, the moral unit of attention is not just the individual but the society.

## Two Epistemic Transitions: From Assets to Capabilities and from Individuals to Systems

If viewing scientific knowledge merely as an asset to which individuals alone are entitled leads to narrow and ultimately inefficient RCB strategies, perhaps it is necessary to re-calibrate our understanding of scientific knowledge and its normative dimensions. This section, therefore, moves the focus from knowledge assets to knowledge capabilities and from individuals to the wider social factors that facilitate or hinder knowledge creation processes, i.e. to institutions and systems.

The term *capability* is perhaps one of the most ubiquitous and ambiguous in the academic literature. It is mainly used in the business literature as *organisational capabilities* to refer to those intangible assets that enable organisations to manage resources and gain a competitive advantage (Ulrich and Smallwood 2004). Perhaps more relevant to the present discussion, the notion of capability is also used (although less extensively) in the economic literature, notably by Abramovitz (1986), who coined the notion of *social absorptive capabilities*: people’s technological competences (loosely measured by years of education) and the social, economic and political institutions that influence those competences. For Abramovitz, the rate of technological convergence (the speed at which less technologically developed countries *catch up* with those more technologically advanced) depends on the social capabilities of a nation. Similar to Abramovitz, Furman et al. (2002) also apply the term at the country level to explain countries’ differential abilities to innovate and commercialise new technologies (*National Innovation Capability* theory). National innovation capabilities do not depend solely on a country’s innovation infrastructure and related outputs, but fundamentally on the environment that determines the innovation process, particularly public policy (e.g. regarding research expenditure, commercialisation, etc.). These different theories point to a notion of capabilities as *the ability of firms/countries to do something worthwhile*, a process enabled by having access to knowledge assets and influenced by external social or political factors. However, these theories do not explain knowledge creation processes in the first place and above all, because they are mostly descriptive, they do not assist with the normative evaluation of arrangements for the creation of knowledge.

The notion of capabilities proposed by Sen (2001) allows shifting the informational basis from command over knowledge assets to the ability to produce those assets. Sen’s Capabilities Approach (CA), however, is firmly rooted in the individual, although many have expanded it to acknowledge the existence of social (collective) capabilities (Evans 2002; Stewart 2005; Deneulin 2008; Ibrahim 2006; Fernández-Baldor et al. 2012). It is from this notion of social capabilities that the CA represents the most appropriate framework to articulate the moral relevance of scientific knowledge and evaluate the social and economic arrangements that impact on societies’ ability to produce it. The remaining sections of this article therefore outline the CA and the rationale for including social capabilities. The article then places scientific knowledge within the domain of social capabilities and sketches some of the implications for RCB.

### The Capabilities Approach and its Social Dimension

The CA is an evaluative framework for the assessment of individual wellbeing and social arrangements, and for this reason it is widely used in the design of policies. For the CA, social and economic development is about enlarging what people can be and do, in contrast with other development paradigms that focus on maximising utility or satisfying basic needs by supplying essential commodities (Fukuda-Parr 2003). The CA shifts the evaluation of development from the commodities people have or lack to the opportunities open to them. This is obviously relevant for the evaluation of technological progress, which does not depend as much on access to information assets (scientific publications, etc.) as on the capacity to produce locally and socially valuable scientific knowledge, as already argued.

Central to the CA is the notion of *capabilities* and *functionings*: capabilities are the real opportunities open to individuals (and, as it will be discussed below, societies) to realise different functionings or achievements that they recognise as important. Capabilities, thus, refer to a particular conception of freedom as the ability to achieve the kind of life one has reason to value. Capabilities are what is effectively possible given individuals’ internal traits and external conditions; functionings are what is actually realised. This distinction is important, as it sets the CA apart from other theories of justice that consider the distribution of utilities (Robbins 1933), primary goods (Rawls 1971), or resources (Dworkin 1981) of intrinsic moral importance. For these approaches only means/resources are inherently valuable; non-material considerations are of no moral relevance. Thus, access to scientific publications, removing IP protections or participating in equitable research partnerships are the ends of distributive justice, but without taking full account of the factors affecting the ability of societies to convert these *goods* into useful scientific knowledge. By focusing on capabilities as ends, the CA acknowledges the existence and moral relevance of material and non-material constraints to development, i.e. the forces that help or hinder one’s capacity to convert capabilities into functionings, opportunities into achievement. In Sen’s terminology, these are *conversion factors*.

A key feature of the CA is its strong moral individualism, which emphasises individuals as the sole subjects of moral concern. For Sen, states of affairs must be evaluated only by their effect upon individuals. This is not to say that the CA does not recognise the importance of groups, institutions and other social arrangements (*collectives*) in enhancing or hindering individual freedoms; however, *their roles can be sensibly evaluated in the light of their contributions to our freedom* (Sen 2001), i.e. as conversion factors. Thus, for Sen, *collectives* enter the evaluative space only insofar they affect individual wellbeing. Many, however, disagree with such instrumentalisation (e.g. Evans 2002; Ibrahim 2006; Stewart 2005; Deneulin 2008; Fernández-Baldor et al. 2012), arguing that *collectives* are not just a means for realising individual freedoms; they are constitutive to those freedoms.

Asserting the constitutive importance of *collectives* rests upon a fundamentally relational conception of individual freedom: a social phenomenon defined against its specific historic, social or political context (Otano-Jiménez 2015). The individual focus of the CA offers a robust defence of individual freedom but cannot help to identify the processes necessary to promote those freedoms. A relational conception of freedom, instead, provides an analytical lens to understand social commitment to individual freedom. Here, the starting point of analysis is not so much the individual but the forms of solidarity that enable the expansion of individual capabilities through the establishment of just social institutions *(ibid)*. Such a broadened focus requires ancillary concepts such as *social capabilities*: capabilities that can only be achieved by individuals through their participation in social institutions (*collectives*). Social capabilities emerge from the exercise of collective agency in ways that are more than the sum of individual capabilities (Stewart 2005), and their benefits cannot be achieved by individuals alone (Ibrahim 2006).

The idea of social capabilities remains contested (Robeyns 2005; Alkire 2008; Cleaver 1999). A key concern is that any attempt to move the focus away from the individual may overlook the dynamics of inequality and exploitation within groups/societies that may negatively impact upon individual freedoms (Cleaver 1999). Social capabilities enable the achievement of goals that cannot be realised by individuals alone but can also lead to exclusion (e.g. ethnic discrimination) or bring about negative consequences for individuals (e.g. oppression of women or minorities within groups). Thus, while some see *collectives* as enabling and intrinsic to human flourishing (Ibrahim 2013) others see them as potentially repressive and thus instrumentally valuable only insofar they do not oppress individual agency. In other words, both Sen and his critics recognise the importance of *collectives* and their relationship with individual freedom, but they disagree (1) on the nature of this relationship—instrumental or intrinsic-, and (2) their potential to oppress or enhance those freedoms. Before proceeding to consider S&T as a collective capability, let us briefly address these two disagreements.

The rationale for considering *collectives* intrinsically valuable beyond their contribution to the lives of individuals can be found in the concept of *irreducibly social goods* (Taylor 1995): goods that cannot be reduced to individual acts or choices since those acts and choices are only possible through collective agency. Language and culture are paradigmatic examples of irreducibly social goods, as they cannot be reduced to individual utterances but only exist within a set of shared norms and codes shaped by collective agency. For Gore (1997), institutional arrangements are also irreducibly social goods, since they are the codes and practices that *constrain and enable human activity, and at the same time they are themselves constituted through that activity (ibid).* Irreducibly social goods, such as language, culture, institutional arrangements and, as it will be argued shortly, knowledge, cannot come into being through individual agency (they are not the goods *of* individuals but *of* society). They have value beyond the individual because they do not benefit individuals but society as a whole (they are not goods *for* individuals but *for* society). Failing to recognise their intrinsic value by incorporating them in the evaluation of development only as instrumental to individual wellbeing is failing to recognise the intimate connection between the individual and society. Acknowledging social goods as intrinsic to individual wellbeing adds an important layer to the evaluation of states of affairs. This point is important: it does not mean that individual wellbeing should be subsumed within *collectives* but that both should enter the evaluative domain.

If irreducibly social goods are constituted through the activities of individuals in ways that are more than the sum of the parts, the social capability to produce such goods is also constituted through the capabilities of individuals in ways that are more than just the sum of individual capabilities. For example, the capability for democratic processes depends upon individuals having the freedom to vote and express their views without fear, yet cannot be reduced to these individual freedoms: it requires concerted action. Social capabilities, thus, *emerge* from the interconnected actions of individuals (and their capabilities) within societies. Collective capabilities do not exist without individual capabilities. At the same time, individual freedoms can only be understood against the collective capability that enables them.

There remains of course the issue of inequality and oppression. Proponents of social capabilities have yet to provide a satisfactory solution to the tension between the individual and the collective, for a focus on the latter can obscure internal dynamics of inequity that oppress individual freedoms (for example, when empowering groups suppresses minority voices or leads to inequities in the way interests are aggregated). However, the same holds for the individualistic view too, for example, when enhancing one capability leads to inequality with regards to other capabilities. One may endorse compulsory primary school education because being able to read and write is a basic capability. Yet, in poor societies, this may disproportionately affect households that critically depend on child labour for their subsistence (another basic capability). Thus, when capabilities conflict, a focus on the individual does not necessarily lead to enhancement of individual freedoms. On the other hand, the inextricable link between social and individual capabilities means that the more the latter are enhanced, the more the former are empowered, and vice-versa. In other words, a well-functioning society is only possible when all individuals are empowered through equality of opportunity. At the same time, individual empowerment necessitates the existence of strong social institutions. Acknowledging the importance of social capabilities need not be acritical but requires an evaluative framework to determine which ones strengthen the process of development and expansion of freedoms and which do not, just as evaluative frameworks are needed to distinguish between good and bad individual capabilities.

### Scientific Knowledge as an Irreducibly Social Good

Despite recognising the importance of S&T for development and its positive and negative impact on political and economic relations within and between countries, much of the S&T literature lacks a normative direction. Normative discourses, on the other hand, have engaged with S&T mostly from the perspective of its potential harms and benefits to individuals. Distributive justice concerns have mostly been raised in the context of access to existing scientific knowledge—see, for example, the work of Pogge (2011) on access to essential medicines—rather than on the capacity to generate such knowledge. This article shifts the focus of analysis from access to knowledge to capabilities, here considered not at the micro level of individual empowerment but at the macro level of systems and institutions strengthening. Such a move is achieved by construing scientific knowledge as an *irreducibly social good*, and the capability to produce it as a social capability that depends upon the existence of adequate social institutions. It is important to consider research capacity holistically and as a currency of justice if S&T policies are to have a substantial and lasting impact on development.

Sen’s CA helps to articulate the claim that equitably sharing in the benefits of S&T requires not so much the distribution of data, information or *existing* codified knowledge but the distribution of the capability to produce and use *new* scientific knowledge (tacit at first and subsequently codified) as a pre-requisite for human and economic development. Such a capability is a social capability because scientific knowledge is an irreducibly social good: it cannot be reduced to individual acts of learning but is *situated* within a *scientific culture* (codes, institutions and practices) and co-evolves with it. That is, scientific knowledge determines and is determined by its specific social context (e.g. when social values determine what scientific questions count as important, and the answers to those questions in turn shape social values). In this sense, scientific knowledge is an irreducible feature *of* society and not *of* individuals. Scientific knowledge (especially basic or non-applied knowledge) is not instrumental to individual wellbeing and cannot be judged through its effects on individuals since it cannot be directly applied to them (e.g. understanding the relationship between folic acid, mood and cognitive function is of no direct benefit to individuals but can help the scientific community to develop effective treatments for depression or dementia). In this sense, scientific knowledge is a social good, valuable *to* society as a whole insofar it expands its opportunities for developing the processes and applications (vaccines, medicines, etc.) necessary for advancing individual wellbeing.

Thus, if scientific knowledge is an irreducibly social good (more than the sum of individual research efforts and benefits society rather than individuals), the capability for knowledge creation is best conceived as a social capability. It creates a critical mass of expertise that is essential for innovation and is valuable to society for its self-realisation. This has a completely different set of implications from an evaluation of knowledge production simply in terms of its contribution to individual capabilities. In the classical Senian approach, the value of scientific knowledge would be relevant only insofar it improves the lives of individuals, i.e. as an *ingredient* of individual human wellbeing. The upshot is that only knowledge that is directly applicable would count as valuable, which automatically disqualifies most of the scientific enterprise. As a social capability, however, the ability to produce scientific knowledge is valuable beyond its actual benefits to single individuals; it expands society’s innovation capital, thus diversifying and widening the range of possible solutions to its specific problems. In other words, scientific knowledge is part of a nation’s intellectual capital (competencies, knowledge, skills) that sustains development. Seen from the perspective of the CA, therefore, scientific knowledge creation becomes part of the capability set that can empower developing societies to redraw the boundaries of development and as such it cannot be construed as an individual good.

There are of course, two important objections to the above argument. First, construing scientific knowledge as a social good valuable *to society as a whole* can mask potential uses in ways that hamper individual wellbeing (e.g. when scientific knowledge is used in warfare), or that advance the wellbeing of certain individuals/groups over others. For example, in highly stratified societies, the production of scientific knowledge may be disproportionately directed towards addressing the health needs of higher socioeconomic groups, thereby neglecting minorities. Yet, while this criticism is a potential limitation of the present argument, it is important to draw a distinction between the capability to produce scientific knowledge (which needs to be evaluated at the level of society) and the application of such knowledge to develop technologies, medicines, etc., that advance (or not) individual wellbeing. As pointed out above, collective capabilities exist alongside individual capabilities as two sides of the same coin. Sen acknowledges the existence of valuable and non-valuable capabilities (Stewart 2005); in the same vein the existence of *good* and *bad* social capabilities can be posited according to how these affect individuals within their societies. Thus, whilst the capability to produce scientific knowledge requires collective empowerment (in the form of policies, institutions, etc.), how such capability is used must be morally evaluated in terms of equitable individual empowerment if the abovementioned issues of discrimination, corruption and nepotism are to be avoided.

Second, construing scientific knowledge as a feature *of* society, i.e. as an endeavour that requires collective agency, does shine a light on the need to strengthen the institutions and structures for knowledge creation through a holistic approach to RCB. However, it can also downplay the critical role that individual agency has in the process of knowledge creation and thus the importance of creating the right set of conditions for individuals to flourish through meaningful and fair participation in the collective production of knowledge (Timmermann 2017). In other words, focusing on institutional strengthening can lead to treating individual scientists mainly as contributors to the process of knowledge production overlooking the fact that they also benefit from it. Though these are important concerns, they stem from positing a false dichotomy between individuals and society. If, as argued before, social capabilities depend upon the existence of individual capabilities and vice-versa, strengthening social institutions requires paying attention to how individuals are empowered and benefit from the production of knowledge. Construing knowledge as a social good calls for a holistic approach to RCB aimed at creating enabling environments for S&T through adequate institutional arrangements. These must include opportunities for individual scientists’ development (e.g. training), as well as incentives that reward collegiality (for example through data sharing), rigour and academic excellence (Rappert and Bezuidenhout 2016). The relationship between individual and social capabilities should not be seen as exclusory but reciprocal. Individuals’ scientific capabilities depend upon social arrangements (e.g. public policies on education, employment, and commercialisation of scientific findings, participation in scientific networks, etc.). They are *socially dependent individual capabilities* (Davis 2015). At the same time, a social capability to produce knowledge crucially rests upon individuals’ commitment to the scientific community and the scientific endeavour. That is, they are *individually dependent social capabilities (ibid)*. For this reason, the dichotomy between individual and social capabilities is fallacious (at least at the theoretical level), for both are interdependent.

On the operational level, however, individual and collective capabilities can clash, for a focus on the collective can obscure inequities in the allocation of resources among members of a scientific community, as well as discriminatory practices resulting in the reinforcement of scientific elites, inequitable access to opportunities for education and training or even exploitation of under-recognised categories of scientists/workers (Timmermann 2017). For a collective capability to produce knowledge can be achieved through institutional arrangements that advantage some and disadvantage others, and a focus on *collective* social institutions, as discussed above, needs to contend with the problem posed by the inevitable aggregation of interests. This is not just a problem for the CA but for public policy in general. However, if knowledge creation is conceived as a capability that empowers societies to pursuit their own self-defined goals, as stated above, the ways in which knowledge is created matter instrumentally. For by fostering diverse and inclusive scientific communities, societies ensure the breeding of a wide range of scientific ideas, which in turn expand the range of possibilities for innovation and consequently development. Thus, the evaluation of social arrangements can be made on the basis of how well or badly they promote the production of the broad base of scientific ideas necessary for innovation and development.

### The Social Capability for Scientific Knowledge: Implications for RCB

Recognising that scientific knowledge is an irreducibly social good whose realisation depends on the existence of social capabilities requires moving the focus of analysis towards what Deneulin (2008) defines as *structures of living together*: a concept originally coined by French philosopher Paul Ricoeur to describe the social institutions within a historical community (people, nation, region): *a structure irreducible to interpersonal relations and yet bound up with these* (Ricoeur 1992). Social institutions, according to these authors, are characterized by *‘a bond of common mores’ (ibid)* from which *power in common*—the capacity to act together—emerges. In other words, social institutions are constituted by individuals bound by common norms, codes and practices in ways that transcend interpersonal relations (Ricoeur refers to the enmeshing of relationships that encompass the plurality of distant others). It is because of this indivisibility that empowerment through collective action is more than the sum of individual efforts. Social structures, therefore, are the locale where empowerment occurs and social goods can be realised, and for this reason they matter beyond their effect upon individuals. From this follows that the production of scientific knowledge by individuals interconnected through common norms and practices is intrinsically bound to the local social structures where those relationships (and the knowledge that emerges from them) are formed. Consequently, an entitlement to the capability to produce scientific knowledge entails a corresponding obligation to assistance to strengthening the necessary processes and institutions, i.e. the connections between actors within the innovation system. This broadens the scope of justice beyond the development of individual capabilities and requires an approach to RCB beyond the individual level.

This article posited scientific knowledge as an *irreducibly social good*: a good that does not belong to individuals (in the sense that it cannot be reduced to individual acts of learning) and has value beyond its contribution to individual wellbeing (in the sense that it does not benefit individuals directly but society as a whole by expanding its opportunities for innovation). Paraphrasing Ricoeur and Deneulin, scientific knowledge emerges from the *structures of knowing together*, that is, from the array of social institutions and the interactions between them. The importance of institutions in the creation of scientific knowledge and innovation is not new but can be traced to the influential concept of National Innovation Systems (NIS) (Freeman 1989; Lundvall 1992), which emphasise the role of institutions in creating and sustaining environments that enable the production and sharing of collective knowledge and resources for the pursuit of social, technological and economic innovation. However, whilst the NIS concept (and its various subsequent derivations) has been useful in highlighting the systemic nature of knowledge production, it is almost exclusively concerned with the commercialisation of innovation, and therefore decisively centred in the firm (Godin 2009), with others institutions (government, universities, industry, non-profit, etc.) providing only a supporting role (Watkins et al. 2015) and being defined by and devoted to this commercialisation end (Godin 2009). Moreover, and paradoxically, the NIS approach does not connect the processes of knowledge creation and diffusion with the political processes of institutional capacity strengthening and governance. The normative approach proposed here bridges this divide; considering knowledge creation processes as a social good on one hand reaffirms the systemic (social) nature of scientific knowledge (a good *of* society), and on the other makes explicit the relationship between scientific knowledge and its ultimate goal: social transformation (a good *for* society). This dual social dimension of scientific knowledge, in turn, brings to the fore the moral importance of social and political institutions (governments, universities and research institutions, scientific societies, funders, patient organisations and other civil society groups, industry, etc.) not just as mere facilitators of knowledge creation but as mutually interdependent enablers and constrainers of behaviours, agendas and ultimately performance. This provides a strong rationale for new approaches to RCB that focus on the strengthening of institutional and governance processes alongside traditional technical skills building.

In practice, this means shifting the focus of RCB initiatives from the individual researcher to the social environment that facilitates or hinders knowledge creation processes. If scientific knowledge is a social good that emerges from the social structures of living and knowing together, a broader approach to RCB is needed, one that moves beyond an almost exclusive focus on individuals and to some extent research infrastructure (Beran et al. 2017) towards creating enabling institutional, organisational and policy environments for the conduct and translation of research and its embedding into public policy. Strengthening capacity at the level of the individual is not optional; on the contrary, workforce development is the backbone of research systems. However, long-term and sustainable research and innovations systems require a multilevel approach that addresses the multiplicity of disabling factors common to most developing countries: technical know-how and resources for sure, but also insufficient ownership of research agendas (still largely dominated by Western donors), geographic isolation and peripheral engagement with the global scientific community, inadequate engagement with users of research (industry, communities and notably policy makers), and above all lack of political buy-in and supportive public discourses (for example, climate change in the case of renewable energies). It has been argued that in most developing countries it is not the lack of a trained workforce or specific technical competencies but the insufficient coordination between the different components of the innovation system that hinders the process of knowledge creation (Arocena and Sutz 2000). Highlighting the normative importance of the *structures of knowing together*, therefore, sheds light on this often-missing relational dimension of knowledge creation.

## Is RCB Neocolonialist**?**

In arguing for an entitlement to a process of development that includes scientific knowledge creation, some may see the threat of neocolonialism. Does RCB endorse, if not impose, a Western paradigm of development grounded in some form of technological determinism, i.e. the assumption that scientific and technological development drives social and human development (Cherlet 2014)? Fully addressing this concern is beyond the more modest aims of this article but below are a few pointers to frame further discussion.

This paper uses an understanding of scientific knowledge (the philosophical worldview, activities, and social institutions that since the Scientific Revolution are identified as modern science) which has historically contributed to a Eurocentric account of progress. Technological progress helped the West portray itself as developed, civilised and rational, in contrast with a rest of the world that was undeveloped, savage and irrational (Harding 1994), thus justifying centuries of colonial domination (Seth 2009). From this perspective, any attempt to build or strengthen scientific research capacity in LMIC may be seen indeed as a neocolonialist imposition. However, without denying that some approaches to RCB may be questionable for their disregard of local agency and values, neocolonialist labels are unhelpful as they preclude more nuanced analyses of broader power and social justice issues (Horton 2013). For example, HIC’s framing of aid (including RCB) in the *national interest* subordinates development priorities to the needs of HIC. This compromises the ability to bring clear benefits to LMIC because it denies them agency and fails to create the open forms of governance and alliances necessary to respond to development challenges. Dismissing RCB as neocolonialist does little to address these issues and to strike the right balance between benefits accrued to HIC and long-term benefits given to LMIC.

RCB, understood as a process of capability expansion, helps create a new balance of power by redrawing the geographies of science. The history of Western science is a history of culturally biased *patterns of systematic knowledge and systematic ignorance* (Harding 1994), for the questions that came to count as *scientific* were those whose answers benefitted colonial powers: improvement of land and sea travel, identification of economically valuable indigenous species, or understanding tropical diseases to maintain the colonies healthy and economically viable (Lock and Nguyen 2010). Other aspects of nature which did not benefit the expansionist West remained uncharted. Because of the still uneven geographic concentration of scientific capacity, many of these biased patterns of knowledge creation have remained even after decolonisation and global scientific priorities (and funding) continue to be established with the tunnel vision of developed countries’ needs. Empowering LMIC to strengthen scientific knowledge production processes can thus help redress the epistemic biases abovementioned by expanding the range of global research actors and, consequently, definitions, priorities and agendas beyond reductionist understandings of progress based on Western-construed categories.

Neither does RCB impose a Western paradigm at the expense of non-Western approaches and forms of knowledge. This implies an artificial epistemological distinction between Western and non-Western knowledge given (a) the diversity within each construct and (b) the fact that what is defined today as traditional non-Western knowledge has been in contact and extensively influenced by Western knowledge for centuries, and vice-versa (Agrawal 1995). Moreover, this article argues for strengthening indigenous S&T capacity, and this presupposes the enmeshing of local knowledge in the scientific enterprise, and a significant degree of control over knowledge-creation processes that precludes any attempt to impose exogenous cultural constructs. Nonetheless, it is important to recognise that indigenisation of scientific knowledge cannot be achieved without a deep engagement with the values and aspirations of the communities such knowledge is intended to benefit (Fejerskov 2017). Although this is implicit in the present argument, a more detailed analysis is required in order to devise effective approaches to community engagement, especially in countries deeply stratified along socioeconomic and ethnic lines.

Finally, RCB does not downplay the considerable research that takes place in LMIC (particularly emerging knowledge economies) but emphasises the need to further shift the geographic boundaries of science. The great scientific contributions of the non-Western world are largely forgotten and need re-appropriation. RCB does not ignore the economic and political agency of LMIC, but acknowledges that the pressures of global market forces and the disruptive effects brought forth by rapid technological change can expand or restrict economic, political and social opportunities (Archibugi and Pietrobelli 2003). Most LMIC recognise with a sense of urgency that these opportunities cannot be fully exploited by simply consuming S&T (Fu et al. 2011; Ghani 2017). The approach to RCB advocated in this article, thus, responds to this recognition.

## Conclusions

Scientific knowledge remains unequally distributed, but more so is the capacity of societies to produce it. Although strengthening the scientific capacity of developing countries is a priority for development cooperation, these efforts are not underpinned by a properly articulated theory of justice. Rather, they seem to rest upon two implicit assumptions: first, that closing the capacity gap requires fairer access to codified knowledge/information; second, that scientific knowledge is a good to be distributed to individuals alone, thus reducing RCB to strengthening scientists’ technical competencies through education and training without parallel investments to develop and sustain the social structures that facilitate knowledge creation.

This article problematises these assumptions by showing the limitations of a focus on the distribution of *existing* knowledge, not always relevant to the needs of LMIC and not always utilisable due to lack of adequate structures for the translation of such knowledge into social and economic development. The CA is therefore used here as a justice framework to move beyond issues of access and command over knowledge assets and articulate the idea that what matters for development is the distribution of the capability to produce knowledge, thus highlighting a moral case for assistance to RCB. Though RCB has been a development priority since the 1990s, a clear understanding of what exactly constitutes research capacity is missing. Consequently, RCB interventions have focused on enhancing individuals’ competencies through education and training, also because these are relatively easier to implement and evaluate. Such approaches, however, have not taken sufficient account of the need to strengthen the social, political and economic structures that connect the different components of a nation’s innovation system. This represents a moral blind spot that masks important questions regarding the economic and political arrangements that help or hinder the scientific divide between rich and poor countries.

Using the concept of *irreducibly social goods* and expanding Sen’s CA approach to include collectives, scientific knowledge is framed as a social good. Consequently, the (social) capability to produce such good requires the strengthening of the social structures for the production of knowledge. This has implications for the interpretation of the human right to science and culture (Article 27 of the UDHR) beyond its current focus on access to scientific knowledge, and for focusing science policy and global research consortia to design holistic approaches to capacity building beyond individual training/skills building.

Scientific capabilities are shaped by country-specific political and institutional contexts, and are thought to reflect countries’ different trajectories of development and patterns of strengths (Bartholomew 1997). Seen from this perspective, scientific development is a local phenomenon rooted in the knowledge, skills, etc. accumulated over time and which constitute a nation’s innovation capital, its *preferred solution* for advancing development. Scientific knowledge as a social good and knowledge creation as a social capability emphasise the importance of construing S&T as spatially and temporally situated, and therefore of paying attention to the unique enmeshing of historic, cultural and social influences that determine the institutional landscape of local research and innovation systems and their functioning. This should warn funding bodies and capacity building experts against the temptation of simply transferring decontextualized blueprints or re-packaging solutions mechanistically—a one-size-fits-all approach. Instead, it calls for more flexible and innovative ways of fostering capacity, beyond simply developing skills so that scientists may fit some pre-defined model, but supporting people, organisations and institutions to challenge current states of affairs and effect change. The idea of social capabilities grounds S&T in its specific social milieu and calls for research leaders and policy makers in LMIC to view capacity development as above all an endogenous and participatory process that requires paying attention to and engaging with society’s specific needs and attitudes (e.g. with regards to emerging technologies). It also challenges them to focus on the bigger picture and shape political agendas from the bottom up. Lastly, it calls for local leaders to challenge the seriously flawed model of capacity building that assumes that external actors know better what their capacity needs are.

The growth of transnational research networks is dissolving national borders, suggesting that S&T is also a global process (Bartholomew 1997) consisting of converging standards and complex governance processes. This may throw into question the relevance, or even possibility, of local research and innovation systems grounded in contextual specificity as argued above. This tension between the local and the global may be resolved by acknowledging the need for differentiated scientific capabilities that on one hand respond to local knowledge needs, and on the other enable synergistic relationships for the tackling of common problems. In this regard, local innovation remains relevant not just because it better serves local demands, but also because it diversifies and widens the range of possible solutions to global technological problems. This provides a powerful incentive for international cooperation and justifies global action for assisting LMIC to strengthen their local research systems.

## Footnotes

1 This is the traditional World Bank classification based on Gross National Income (GNI). It has been suggested to be too broad to be distinctive given it groups together countries with different indicators of development, including scientific capacity. Throughout this article, LMIC refers to countries in the low and lower-middle income distributions and excluding upper middle countries (e.g. Argentina, South Africa and Mexico), which are included in the upper-middle distribution as high-income countries (HIC). Whilst recognising that scientific capacity does not necessarily correlate with GNI, the LMIC–HIC classification was chosen for simplicity, and on the basis that overall most countries along the low-middle income distribution tend to underinvest in science and technology, compared to countries in the upper and high-income distributions (Rabesandratana 2015), although this is not an absolute rule.

2 Whilst some commentators prefer to use the term ‘capacity development’ instead of ‘capacity building’ to emphasize that abilities are strengthened and enhanced, rather than built from scratch (Vallejo and When 2016), in practice there is little operational difference between the two terms. Therefore, ‘capacity building’ is used here as equivalent to ‘capacity development’.
